# Capacity, committed funding and co-production—institutionalizing implementation research in low- and middle-income countries

**DOI:** 10.1093/heapol/czaa120

**Published:** 2020-11-06

**Authors:** Jeanette Vega, Zubin Cyrus Shroff, Kabir Sheikh, Irene Akua Agyepong, Binyam Tilahun, Viroj Tangcharoensathien, Assad Hafeez, Indu Bhushan, Abdul Ghaffar, David Peters

**Affiliations:** 1 Red de Salud UC-Christus, Chile; 2 Alliance for Health Policy and Systems Research, WHO, Geneva, Switzerland; 3 Ghana Health Service Research and Development Division, Accra, Ghana; 4 University of Gondar College of Medicine and Health Sciences, Gondar, Ethiopia; 5 International Health Policy Programme, Thailand; 6 Health Services Academy, Islamabad, Pakistan; 7 National Health Authority, New Delhi, India; 8 Department of International Health, Johns Hopkins Bloomberg School of Public Health, Baltimore, USA

Catalysed by the recognition that gaps in the implementation and scale-up of proven interventions are a major hindrance to the achievement of universal health coverage (UHC) goals, the field of implementation research (IR) has grown in prominence and importance in recent years (Peters *et al.*, 2013). Several global health programmes and organizations have supported the growth and uptake of IR in low- and middle-income countries (LMICs), reflected in increased funding and an increasing volume of IR publications focused on the delivery of public health policies and programmes (WHO, 2017). However, for IR to achieve its true potential and consistently and systematically impact health policy and programme implementation in LMICs, it needs to become mainstreamed and made routine in those countries—or in other words, institutionalized (Novotná *et al.*, 2012; Koon *et al.*, 2020). 

Unfortunately, in many LMICs, applied and interdisciplinary research is often neglected and tends to be underdeveloped (Acharya and Pathak, 2019; Dobbie *et al.*, 2019) —and IR is no exception. One reflection of this is the fact that a significant proportion of IR produced on LMICs is still produced by high-income country (HIC) based first authors (WHO, 2017). However, at the same time, there are several instances of positive progress towards institutionalizing IR in LMICs and in this commentary, we elaborate on some of those experiences. Based on these experiences, we submit there are three core pre-requisites for the institutionalization of IR in LMICs each deserving equal focus and attention—namely, capacity-building, committed funding and co-production. We describe the steps that some LMICs have taken in each of these areas and draw lessons for other LMICs to consider.

In the first place, institutionalization necessitates adequate capacity for the generation of relevant and useful research, and research institutions with capacities to manage and ensure the quality of IR. Thailand is a key exemplar of country commitment to develop research capacity in IR. The International Health Policy Programme (IHPP) has since 2001 maintained a programme to award fellowships to promising young public health professionals train as researchers. Fellows are required to be mentored for up to two years by senior researchers prior to being placed at Masters or PhD programmes at world-leading institutions. The fellows have played a crucial role in contributing to Thailand’s IR capacity after their return (Pitayarangsarit and Tangcharoensathien, 2009). A distinct approach that can be found in a more recent initiative is COMCAHPSS (The Consortium for Mothers, Children, Adolescents and Health Policy and Systems Strengthening), a South-South research capacity strengthening initiative that brings together 19 institutions from nine countries in the region. In this regionally focused, network-based approach, capacity strengthening has centred around the provision of pre-doctoral training in research methods for early to mid-career researchers from the region. Additionally, the initiative has also supported early career researchers secure PhD admissions through the provision of funding support, by facilitating access to other sources of funding and establishing mentoring links between students and prospective supervisors (Barasa *et al.*, 2019).

The continuous generation of a steady stream of research also requires committed and regular funding (Panisset *et al.*, 2012; Peters *et al.*, 2013; Shroff *et al.*, 2017). In far too many LMICs, IR continues to be project based and driven by actors who are not invested in local contexts, leading to research that does not serve local and country priorities to the extent that it should (WHO, 2017). In particular, LMIC-based institutions engaged in IR face considerable challenges obtaining sufficient and flexible funding (Shroff *et al.*, 2017). Ensuring the availability of committed funding for IR has a major role in enabling institutions to develop research priorities responsive to local contextual needs (Shroff *et al.*, 2017). This challenge has been well recognized by some national governments such as Thailand and India which have committed significant resources to IR through investments in the IHPP and National Knowledge Platform, respectively (Pitayarangsarit and Tangcharoensathien, 2009; Sheikh *et al.*, 2016).

Finally, IR is applied research, and as such it also requires establishing mechanisms to promote and facilitate the development of meaningful collaborations between researchers and implementers. Arrangements that allow researchers to form teams and collaborations with each other and with implementers, to win research grants, and to have the benefits and support of institutional affiliation as they progress in their careers are crucial enablers to IRs institutionalization. Over the past few years, the University of Gondar in Ethiopia has collaborated with Ethiopia’s Federal Ministry of Health in managing national IR programmes on themes of national importance (immunization services, health workforce policy), providing technical assistance to research teams comprising of researchers and implementers across the country. Another such experience is that of Pakistan’s Health Services Academy, which has collaborated with policymakers in managing countrywide IR programmes on immunization and is currently managing IR to inform the implementation of Pakistan’s National Health Insurance *Sehat Sahulat* Scheme. In India, the National Health Authority (NHA) has engaged with WHO and several leading Indian research institutions in a programme of IR on India’s National Health Insurance Scheme (PM-JAY). Learning events focused on the interpretation and dissemination of research findings brought together implementers and researchers in strategizing how to improve the performance of the scheme.

Capacity-building, mechanisms for research co-production and funding for IR are synergistic investments that can enable IR to achieve its unrealized potential to help countries move towards UHC. Each is critical for effective institutionalization, yet none are sufficient in themselves. In each of the experiences cited, leadership has been a key factor in the articulation of a comprehensive vision for the institutionalization of IR and the perseverance to establish the appropriate mechanisms to enact it (Chunharas and Davies, 2016; Koon *et al.*, 2020). For this to happen, countries must develop a comprehensive vision that marries these three types of investments, and create advocacy platforms that help sustain the political commitment for IR. The gains, by way of more responsive, effective and high-quality programme delivery and policy implementation, will speak for themselves.

*Ethical approval.* No ethical approval was required for this study.
